# Brain tumor classification based on neural architecture search

**DOI:** 10.1038/s41598-022-22172-6

**Published:** 2022-11-10

**Authors:** Shubham Chitnis, Ramtin Hosseini, Pengtao Xie

**Affiliations:** 1 Department of Chemical Engineering, Indian Institute of Technology Bombay, Mumbai, India; 2 Department of Electrical and Computer Engineering, University of California San Diego, San Diego, USA

**Keywords:** Cancer imaging, Machine learning

## Abstract

Brain tumor is a life-threatening disease and causes about 0.25 million deaths worldwide in 2020. Magnetic Resonance Imaging (MRI) is frequently used for diagnosing brain tumors. In medically underdeveloped regions, physicians who can accurately diagnose and assess the severity of brain tumors from MRI are highly lacking. Deep learning methods have been developed to assist physicians in detecting brain tumors from MRI and determining their subtypes. In existing methods, neural architectures are manually designed by human experts, which is time-consuming and labor-intensive. To address this problem, we propose to automatically search for high-performance neural architectures for classifying brain tumors from MRIs, by leveraging a Learning-by-Self-Explanation (LeaSE) architecture search method. LeaSE consists of an explainer model and an audience model. The explainer aims at searching for a highly performant architecture by encouraging the architecture to generate high-fidelity explanations of prediction outcomes, where explanations’ fidelity is evaluated by the audience model. LeaSE is formulated as a four-level optimization problem involving a sequence of four learning stages which are conducted end-to-end. We apply LeaSE for MRI-based brain tumor classification, including four classes: glioma, meningioma, pituitary tumor, and healthy, on a dataset containing 3264 MRI images. Results show that our method can search for neural architectures that achieve better classification accuracy than manually designed deep neural networks while having fewer model parameters. For example, our method achieves a test accuracy of 90.6% and an AUC of 95.6% with 3.75M parameters while the accuracy and AUC of a human-designed network—ResNet101—is 84.5% and 90.1% respectively with 42.56M parameters. In addition, our method outperforms state-of-the-art neural architecture search methods.

## Introduction

Brain tumor, where abnormal brain cells grow in an uncontrollable way, is a life-threatening disease that causes about 0.25 million deaths worldwide in 2020^1^. The 5-year survival rate for people with brain tumors is about 36% and the 10-year survival rate is about 31%^1^. Brain tumors vary from non-cancerous benign variants to much more harmful malignant ones^2^. The World Health Organization (WHO) has assigned grades^3^ (I-IV) to tumors based on their severity and other molecular characteristics. Higher-grade tumors are more malignant, rendering patients to have smaller survival rates^2^. Timely diagnosis and treatment is crucial for improving survival rate^2^. Magnetic Resonance Imaging (MRI) is frequently used in clinical practice for identifying the existence and types of brain tumors, due to its noninvasive nature, being less harmful to human bodies, the ability to capture high-resolution images, and the timeliness in getting results^4^. Detecting brain tumors and determining their types from MRI is a highly challenging medical task for physicians, which requires many years of training and medical practice^4^. In medically less developed regions such as rural areas, physicians who can accurately interpret MRI images to diagnose and assess the severity of brain tumors are highly lacking^4^.

To address this problem, artificial intelligence methods (especially deep learning methods)^4–8^ have been developed to provide physicians with decision support for brain tumor classification. In these methods, deep neural networks are manually designed by human experts, which is time-consuming and labor-intensive. For example, to design an effective deep network that is tailored to the unique properties of brain tumor MRI images, human experts need to specific the number of layers in the network, design what operations (e.g., separable convolution, dilated convolution, max pooling, batch normalization, etc.) to use in each layer, specify hyperparameters of operations (e.g., kernel size of convolutions), and so on. The decision space is very large and humans need to spend a lot of time to find out the optimal design. To address this problem, we study how to automatically search for high-performance neural architectures to classify brain tumors, with minimal intervention from humans. Neural architecture search (NAS)^9–13^ has been studied previously. Existing NAS methods are limited in that they are either computationally expensive or cannot search for high-performance architectures. Reinforcement learning^9,11^ and evolutionary algorithms^14,15^ based NAS methods are computationally expensive. While differentiable NAS methods^12,16^ are computationally efficient, their performance is not stable and their searched architectures often perform less well than human-designed architectures. For example, several works^17–19^ have shown that existing differentiable NAS methods are prone to performance collapse: searched architectures perform well on validation data but poorly on test data.

To address the limitations of existing NAS methods, we leverage a Learning-by-Self-Explanation (LeaSE) differentiable architecture search method to automatically search for high-performance neural architectures to accurately and efficiently classify brain tumors^20^. Our method is featured with an explanation-driven search mechanism: an explainer improves its architecture by encouraging the architecture to generate high-fidelity explanations of prediction outcomes, where explanations’ fidelity is evaluated by the audience model. Thanks to this mechanism, architectures searched by our method outperform those searched by state-of-the-art NAS baselines, as shown in experiments. In our framework, both the explainer model and audience model learn to perform MRI-based tumor classification. The explainer has a learnable architecture and a set of learnable network weights. The audience has a predefined architecture and a set of learnable network weights. The goal is to help the explainer search for a well-performing neural architecture. The way to achieve this goal is to encourage the explainer to give clear explanations to the audience regarding how predictions are made. Intuitively, if a model can explain prediction outcomes well, it must have a deep understanding of the prediction task and can learn better based on this understanding. The learning is organized into four stages. At the first stage, the explainer trains its network weights by minimizing the prediction loss on its training dataset, with its architecture fixed. At the second stage, the explainer uses its model trained at the first stage to make predictions on the training data examples of the audience and leverages an adversarial attack^21,22^ approach to explain prediction outcomes. At the third stage, the audience model combines its training examples and the explainer-made explanations of prediction outcomes on these examples to train its network weights. At the fourth stage, the explainer updates its neural architecture by minimizing its validation loss and the audience’s validation loss. The four stages are synthesized into a unified four-level optimization framework where they are performed jointly in an end-to-end manner. Each learning stage influences other stages. Our framework is applied for classifying brain tumors from MRI images. The dataset used in our experiments contains 3264 MRI images from four classes: glioma, meningioma, pituitary tumor, and healthy. Our method achieves better classification accuracy with fewer model parameters compared with manually designed neural networks and previous neural architecture search methods.

The major contributions of this paper are as follows:

- To our best knowledge, our work represents one of the first few works studying automated neural architecture search for brain tumor diagnosis from MRI images. It can automatically search for high-performance neural architectures that achieve state-of-the-art (SOTA) performance in classifying brain tumors from MRIs. Our method can save time cost and labor cost by avoiding manually designing neural architectures.
- Our method is featured with a new Learning-by-Self-Explanation mechanism. An explainer model improves its neural architecture by generating sensible explanations of prediction outcomes, where the sensibility of explanations is evaluated via an audience model. The LeaSE formulation is based on multi-level optimization, consisting of four levels of nested optimization problems which correspond to four learning stages: 1) the explainer trains its network weights; 2) the explainer generates explanations using its trained weights; 3) the audience is trained using generated explanations; and 4) the explainer improves its architecture by minimizing audience’s validation loss. The four stages are performed end-to-end. An efficient gradient-based algorithm is developed to solve the optimization problem of LeaSE.
- Thanks to the Learning-by-Self-Explanation mechanism, the architectures searched by our method not only outperform architectures searched by SOTA neural architecture search baselines and outperform SOTA deep neural networks manually designed by humans in brain tumor classification, but also have fewer weight parameters and smaller model size. On a brain tumor dataset with 3264 MRI images and four classes, our searched architecture achieves a test accuracy of 90.6% and an AUC of 95.6% with 3.75 M parameters, while the accuracy and AUC of a human-designed network—ResNet101—is 84.5% and 90.1% respectively with 42.56M parameters.

## Related works

### Brain tumor classification and segmentation

A variety of deep learning methods^23^ have been proposed for brain tumor classification and segmentation. Menze et al.^24^ developed a multi-modal brain tumor image segmentation benchmark, where 20 tumor segmentation algorithms were evaluated on 65 multi-contrast MRI images that have low-grade and high-grade glioma. Pereira et al.^25^ utilized convolutional neural networks for brain tumor segmentation in MRI images. Havaei et al.^26^ proposed a convolutional neural network for brain tumor classification, which exploits both local features and global contextual features. Afshar et al.^5^ utilized capsule networks to perform brain tumor classification. Chen et al.^27^ proposed a dual-force convolutional neural network for brain tumor segmentation, which leverages multi-level information and a dual-force training mechanism to improve latent representations. Sajjad et al.^28^ utilized deep CNN with data augmentation for multi-grade brain tumor classification. Kaldera et al.^29^ utilized faster region-based convolutional neural networks for brain tumor classification and segmentation. Ghosal et al.^30^ utilized a squeeze and excitation ResNet model for brain tumor classification. Mzoughi et al.^31^ proposed a multi-scale three-dimensional convolutional neural network for glioma brain tumor classification based on the whole volumetric T1-Gado MRI sequence. Pei et al.^32^ proposed a 3D context aware deep learning method for brain tumor segmentation, subtype classification, and survival prediction. Ghassemi et al.^7^ pretrained a deep neural network as the discriminator of a generative adversarial network (GAN) for extracting robust features, which is utilized for classifying brain tumors. Shaik et al.^33^ proposed a multi-level attention mechanism for brain tumor recognition, where spatial and cross-channel attention is utilized to identify tumor regions and maintain cross-channel temporal dependencies. Hao et al.^6^ proposed a transfer learning based active learning method for brain tumor classification. This method aims to reduce human annotation cost and stabilize model performance. Lu et al.^34^ proposed data distillation and augmentation methods for brain tumor detection. This method distills representative examples which are mixed to create augmented examples. Deepak et al.^35^ leveraged a siamese network and a neighborhood analysis method for brain tumor classification. Díaz-Pernas et al.^8^ utilized a multiscale convolutional neural network for brain tumor classification and segmentation. In these existing methods, deep neural networks for brain tumor classification and segmentation are manually designed by human experts, which is very time-consuming and labor intensive. In contrast, our method automatically searches for high-performance neural architectures for brain tumor classification with minimal intervention from humans, which can greatly save time and labor costs in designing deep networks. Neural architecture search (NAS) for brain tumor classification has not been well-explored. To our best knowledge, there are very few related works in this field. Wang^36^ developed an NAS method for gliomas segmentation from multimodal magnetic resonance images. Milesi et al.^37^ applied differentiable NAS for brain tumor segmentation in MRIs. Different from these two works which focus on segmentation, our work focuses on classification.

### Neural architecture search

In the past few years, a wide variety of NAS methods have been proposed and achieved considerable success in automatically identifying highly performant architectures of neural networks for the sake of reducing the reliance on human experts. Early NAS approaches^9–11^ are mostly based on reinforcement learning (RL), which use a policy network to generate architectures and evaluate these architectures on a validation set. Validation losses are used as rewards to optimize the policy network and train it to produce high-quality architectures. While RL-based approaches achieve the first wave of success in NAS research, they are computationally very expensive since evaluating the architectures requires a heavy-duty training process. This limitation renders RL-based approaches not applicable for most users who do not have enough computational resources. To address this issue, differentiable search methods^12,16,38^ have been proposed, which parameterize architectures as differentiable functions and perform search using efficient gradient-based methods. In these methods, the search space of architectures is composed of a large set of building blocks where the output of each block is multiplied with a smooth variable indicating how important this block is. Under such a formulation, search becomes solving a mathematical optimization problem defined on importance variables where the objective is to find an optimal set of variables that yield the lowest validation loss. This optimization problem can be solved efficiently using gradient-based methods. Differentiable NAS research is initiated by DARTS^12^ and further improved by subsequent works such as P-DARTS^39^, PC-DARTS^40^, etc. P-DARTS^39^ grows the depth of architectures progressively in the search process. PC-DARTS^40^ samples sub-architectures from a super network to reduce redundancy during search. While computationally efficient, differentiable NAS methods often suffer the problem of performance collapse^17–19^. Their searched architectures perform well on validation data but poorly on test data. Our LeaSE framework is orthogonal to existing NAS methods and can be used to improve any differentiable NAS method^20^, by encouraging architectures to generate sensible explanations and using auxiliary models to evaluate the sensibility of generated explanations. Such et al.^13^ proposed a Generative Teaching Network (GTN), which learns a generative model to generate synthetic examples and uses synthetic examples to search for the architecture of an auxiliary model. LeaSE differs from GTN in that: 1) LeaSE focuses on searching the architecture of a primary model (the explainer) by letting it explain to an auxiliary model (the audience) while GTN focuses on searching the architecture of the auxiliary model; 2) LeaSE’s primary model produces explanations via adversarial attack while the generative model in GTN generates synthetic examples. Besides RL-based approaches and differentiable NAS approaches, another paradigm of NAS methods^14,15^ are based on evolutionary algorithms. In these methods, architectures are formulated as individuals in a population. High-quality architectures produce offspring to replace low-quality architectures, where the quality is measured using fitness scores. Similar to RL-based approaches, these methods also require considerable computing resources.

## Methods

In this section, we first review differentiable architecture search (DARTS)^12^, then introduce the Learning by Self-Explanation (LeaSE) framework^20^, and finally present an optimization algorithm for LeaSE.

**Figure 1:**
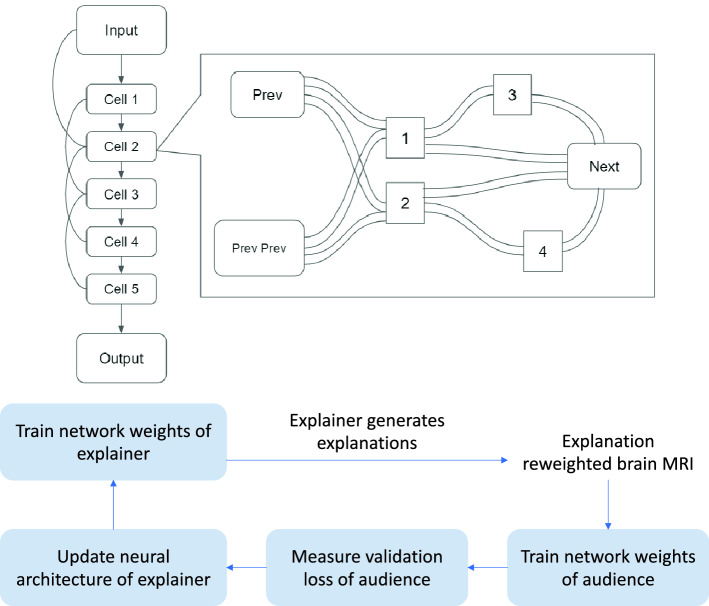
(Top) Search space of DARTS. (Bottom) Overview of the LeaSE framework.

### Differentiable architecture search (DARTS)

Given a predictive task and labeled data, DARTS^12^ aims to automatically search for the optimal neural architecture that can best fulfill the predictive task in a differentiable way. This problem can be formulated as follows:

1 $$\begin{aligned} \begin{array}{ll} \text {min}_A &{} L(D^{\text {(val)}},A, W^*(A)) \\ s.t. &{} W^*(A)=\text {argmin}_{W}\;\;L(D^{\text {(tr)}},A, W) \end{array} \end{aligned}$$

where $D^{\text {(tr)}}$ and $D^{\text {(val)}}$ denote training data and validation data respectively. *A* denotes a neural architecture and *W* denotes model weights. Given a configuration *A* of the architecture, its weights *W* are trained on the training data and the best weights are denoted by $W^*(A)$. The loss $L(D^{\text {(val)}},A, W^*(A))$ of the trained model is measured on the validation set. The goal of DARTS is to identify the best *A* that yields the lowest validation loss. The search space of DARTS (as shown in Figure 1(top)) is defined as follows: set up an overparameterized network which consists of a stack of cells; each cell combines many different types of operations; each operation is associated with an architecture variable (AV) indicating how important the operation is; optimize these AVs together with weight parameters in the operations to achieve the best performance on the validation set; operations with top-*K* largest AVs are selected to form the final architecture. A neural architecture can be represented as a directed acyclic graph (DAG) where nodes represent intermediate representations (e.g., feature maps in CNNs) and edges represent operations (e.g., convolution, pooling) over nodes. Each node $x_i$ is calculated in the following way: $x_i=\sum _{j\in P_i} e_{ji} (x_j)$, where $P_i$ is a set containing the ancestor nodes of *i*. $e_{ji}(\cdot )$ denotes an operation associated with the edge connecting *j* to *i*. In differentiable NAS, this DAG is overparameterized: the operation $e_{ji}(\cdot )$ on each edge is a weighted combination of all possible operations. Namely, $e_{ji}(x)=\sum _{m=1}^{M} \frac{\exp (a_{jim})}{\sum _{l=1}^{K}\exp (a_{jil})}o_m(x)$, where $o_m(\cdot )$ is the *m*-th operation (parameterized by a set of weights) and *M* is the total number of operations. $a_{jim}$ is an architecture variable representing how important $o_m(\cdot )$ is. In the end, the prediction function of this neural network is a continuous one parameterized by the variables $A=\{a\}$ representing the architecture and the weight parameters *W*. The prediction loss function is end-to-end differentiable w.r.t both *A* and *W*, which can be learned by gradient descent. After learning, operations with top-*K* largest architecture variables are retained to form the final architecture. Please refer to Table 1 for notations.

**Table 1:** Notations in learning by self-explanation.

Mathematical notation	Notation’s meaning
*A*	Explainer’s architecture
*E*	Explainer’s network weights
*W*	Audience’s network weights
$\delta$	Explanations
$D_{e}^{(\text {tr})}$	Explainer’s training data
$D_{e}^{(\text {val})}$	Explainer’s validation data
$D_{a}^{(\text {tr})}$	Audience’s training data
$D_{a}^{(\text {val})}$	Audience’s validation data

### Learning by self-explanation (LeaSE)

In this section, we introduce the Learning by Self-Explanation (LeaSE) method which is based on four-level optimization^20^. In the LeaSE framework (as shown in Figure 1(bottom)), there is an explainer model and an audience model, both of which learn to perform image classification (with *K* classes). The primary goal of LeaSE is to search for a well-performing neural architecture for the explainer. The way to achieve this goal is to let the explainer make meaningful explanations of prediction outcomes. The intuition behind LeaSE is: to correctly explain prediction results, a model needs to learn to understand the classification task very well. The explainer has a learnable architecture *A* and a set of learnable network weights *E*. The audience has a pre-defined neural architecture (by human experts) and a set of learnable network weights *W*. The learning is organized into four stages.

At the first stage, the explainer trains its network weights *E* on its training dataset $D_e^{(\text {tr})}$, with the architecture fixed:

2 $$\begin{aligned} E^*(A) =\text {argmin}_{E} \; L(E,A,D_e^{(\text {tr})}). \end{aligned}$$

To define a training loss *L*, it is needed to use the architecture *A* together with network weights *W* to make predictions on training examples. However, *A* cannot be updated by minimizing the training loss. Otherwise, a trivial solution of *A* will be yielded: *A* is very large and complex that it can perfectly overfit the training data but will make largely incorrect predictions on novel data examples. Note that $E^*(A)$ is a function of *A* since $L(E,A, D_e^{(\text {tr})})$ is a function of *A* and $E^*(A)$ depends on $L(E, A,D_e^{(\text {tr})})$.

At the second stage, the explainer uses the trained model $E^*(A)$ to make predictions on the input training examples $D_a^{(\text {tr})}$ of the audience and explain prediction outcomes. Specifically, given an input image *x* and a predicted class label *y*, the explainer aims to find a subset of image patches *P* in *x* that are mostly correlated with *y* and uses *P* as explanations for *y*. LeaSE leverages an adversarial attack based approach^21,22^ to achieve this goal. Adversarial attack adds small random perturbations $\delta$ to pixels in *x* so that the prediction outcome on the perturbed image $x+\delta$ is no longer *y*. Pixels that are perturbed more have higher correlations with the prediction outcome *y* and can be used as explanations. This process amounts to solving the following optimization problem:

3 $$\begin{aligned} \begin{array}{ll} \{\delta ^*_i(E^*(A))\}_{i=1}^N =&{}\text {argmax}_{\{\delta _i\}_{i=1}^N} \;\; \sum _{i=1}^N \ell (f(x_i+\delta _i;{E^*(A)}),f(x_i;{E^*(A)})) \\ &{} s.t. \qquad \quad \;\; \forall i, \Vert \delta _i\Vert _{\infty }\le \tau \end{array} \end{aligned}$$

where $\delta _i$ is the perturbation added to image $x_i$ and *N* is the number of training images. $\tau$ is a small positive scalar. $f(x_i+\delta _i;{E^*(A)})$ and $f(x_i;{E^*(A)})$ are the prediction outcomes of the explainer’s network $f(\cdot ;{E^*(A)})$ on $x_i+\delta _i$ and $x_i$. $f(x_i+\delta _i;{E^*(A)})$ and $f(x_i;{E^*(A)})$ are both *K*-dimensional vectors containing prediction probabilities on the *K* classes. $\ell (\cdot ,\cdot )$ is a cross-entropy loss with $\ell (\mathbf {a},\mathbf {b})=-\sum _{k=1}^K b_i\log a_i$. In this optimization problem, the explainer aims to find perturbations for each image so that the predicted outcome on the perturbed image is largely different from that on the original image. The learned optimal perturbations are used as explanations and those with larger values indicate that the corresponding pixels are more important in decision-making. $\delta ^*_i(E^*(A))$ is a function of $E^*(A)$ since $\delta ^*_i(E^*(A))$ is a function of the objective in Eq.(3) and the objective is a function of $E^*(A)$.

At the third stage, given the explanations $\{\delta ^*_i(E^*(A))\}_{i=1}^N$ made by the explainer, the audience leverages them to train its network weights. Since perturbations indicate how important input pixels are, the audience uses them to reweigh the pixels: $x\odot \delta$, where $\odot$ denotes element-wise multiplication. Pixels that are more important are given more weights. The audience trains its network weights on these weighted images:

4 $$\begin{aligned} W^*( \{\delta ^*_i(E^*(A))\}_{i=1}^N ) =\text {argmin}_{W} \;\; \sum _{i=1}^N \ell (f(\delta _i^*(E^*(A))\odot x_i;W),t_i), \end{aligned}$$

where $f(\delta _i^*(E^*(A))\odot x_i;W)$ is the prediction outcome of the audience’s network $f(\cdot ;W)$ on the weighted image $\delta _i^*(E^*(A))\odot x_i$ and $t_i$ is a class label. $W^*( \{\delta ^*_i(E^*(A))\}_{i=1}^N )$ is a function of $\{\delta ^*_i(E^*(A))\}_{i=1}^N$ since $W^*( \{\delta ^*_i(E^*(A))\}_{i=1}^N )$ is a function of the objective in Eq.(4) and the objective is a function of $\{\delta ^*_i(E^*(A))\}_{i=1}^N$.

At the fourth stage, the explainer validates its network weights $E^*(A)$ on its validation set $D^{(\text {val})}_e$ and the audience validates its network weights $W^*( \{\delta ^*_i(E^*(A))\}_{i=1}^N )$ on its validation set $D^{(\text {val})}_a$. The explainer optimizes its architecture by minimizing its validation loss and the audience’s validation loss:

5 $$\begin{aligned} \text {min}_{A} \; L(E^*(A),A, D_e^{(\text {val})}) + \gamma L(W^*( \{\delta ^*_i(E^*(A))\}_{i=1}^N ), D_a^{(\text {val})}), \end{aligned}$$

where $\gamma$ is a tradeoff parameter.

The four stages are integrated into a unified four-level optimization framework, resulting in the following formulation of LeaSE:

6 $$\begin{aligned} \begin{array}{l} \underset{A}{\text {min}} \;\; \; L(E^*(A),A, D_e^{(\text {val})}) + \gamma L(W^*( \{\delta ^*_i(E^*(A))\}_{i=1}^N ), D_a^{(\text {val})})\\ s.t. \;\;\; W^*( \{\delta ^*_i(E^*(A))\}_{i=1}^N ) = \underset{W}{\text {argmin}} \;\; \sum \limits _{i=1}^N \ell (f(\delta _i^*(E^*(A))\odot x_i;W),t_i)\\ \quad \quad \{\delta ^*_i(E^*(A))\}_{i=1}^N =\text {argmax}_{\{\delta _i\}_{i=1}^N} \;\; \sum _{i=1}^N \ell (f(x_i+\delta _i;{E^*(A)}),f(x_i;{E^*(A)})) \\ \qquad \qquad \qquad \qquad \quad \;\;\; s.t. \qquad \quad \;\; \forall i, \Vert \delta _i\Vert _{\infty }\le \tau \\ \quad \quad E^*(A) = \underset{E}{\text {argmin}} \; L(E,A,D_e^{(\text {tr})}). \end{array} \end{aligned}$$

In this framework, there are four optimization problems, each corresponding to a learning stage. From bottom to up, the optimization problems correspond to learning stage 1, 2, 3, and 4 respectively. The first three optimization problems are nested on the constraint of the fourth optimization problem. These four stages are conducted end-to-end in this unified framework. The solution $E^*(A)$ obtained at the first stage is used to generate explanations at the second stage. The explanations $\{\delta ^*_i(E^*(A))\}_{i=1}^N$ obtained at the second stage are used to train the model at the third stage. The solutions obtained at the first and third stage are used to make predictions at the fourth stage. The architecture *A* updated at the fourth stage changes the training loss at the first stage and consequently changes the solution $E^*(A)$, which subsequently changes $\{\delta ^*_i(E^*(A))\}_{i=1}^N$ and $W^*( \{\delta ^*_i(E^*(A))\}_{i=1}^N )$. Following Liu et al.^12^, we perform differentiable search on *A* in a search space composed of candidate building blocks. Searching amounts to selecting a subset of candidate blocks by learning a selection variable for each block. Selection variables indicate the importance of individual blocks and are differentiable.

### Optimization algorithm

An efficient algorithm is developed to solve the LeaSE problem in Eq.(6). Getting insights from Liu et al.^12^, the gradient of $L(E,A, D_e^{(\text {tr})})$ w.r.t *E* is calculated and $E^{*}(A)$ is approximated using a one-step gradient descent update of *E*. The approximation $E'$ of $E^{*}(A)$ is plugged into $\ell (f(x_i+\delta _i;{E^*(A)}),f(x_i;{E^*(A)}))$, resulting in an approximated objective denoted by $O_{\delta _i}$. Then $\delta ^*_i(E^*(A))$ is approximated using a one-step gradient descent update of $\delta _i$ based on the gradient of $O_{\delta _i}$. Next, the approximation $\delta '_i$ of $\delta ^*_i(E^*(A))$ is plugged into $\sum _{i=1}^N \ell (f(\delta _i^*(E^*(A))\odot x_i;W),t_i)$, resulting in another approximated objective denoted by $O_W$. Then $W^*(\{\delta ^*_i(E^*(A))\}_{i=1}^N )$ is approximated using a one-step gradient descent update of *W* based on the gradient of $O_{W}$. Finally, the approximation $E'$ of $E^*(A)$ and the approximation $W'$ of $W^*( \{\delta ^*_i(E^*(A))\}_{i=1}^N )$ are plugged into $L(E^*(A),A, D_e^{(\text {val})}) + \gamma L(W^*( \{\delta ^*_i(E^*(A))\}_{i=1}^N ), D_a^{(\text {val})})$, resulting in the third approximate objective denoted by $O_A$. *A* is updated by descending the gradient of $O_A$. In the sequel, $\nabla ^2_{Y,X}f(X,Y)$ denotes $\frac{\partial f(X,Y)}{\partial X\partial Y}$. Next, we present the details. First of all, $E^{*}(A)$ is approximated using

7 $$\begin{aligned} E'=E - \xi _{e} \nabla _{E}L(E, A, D_{e}^{(\mathrm {tr})}), \end{aligned}$$

where $\xi _{e}$ is a learning rate. Plugging $E'$ into $\ell (f(x_i+\delta _i;{E^*(A)}),f(x_i;{E^*(A)}))$, an approximate objective $O_{\delta _i}= \ell (f(x_i+\delta _i;E'),f(x_i;E'))$ is obtained. Then $\delta _i^*(E^*(A))$ is approximated using a one-step gradient descent update of $\delta _i$ with respect to $O_{\delta _i}$:

8 $$\begin{aligned} \delta '_i=\delta _i - \xi _{\delta } \nabla _{\delta _i} \ell \left(f(x_i+\delta _i;E'),f(x_i;E') \right). \end{aligned}$$

Plugging $\delta '_i$ into $\sum _{i=1}^N \ell (f(\delta _i^*(E^*(A))\odot x_i;W),t_i)$, an approximated objective $O_{W}=\sum _{i=1}^N \ell (f(\delta '_i\odot x_i;W),t_i)$ is obtained. Then $W^*(\{\delta _i^*(E^*(A))\}_{i=1}^N )$ is approximated using a one-step gradient descent update of *W* with respect to $O_{W}$:

9 $$\begin{aligned} W'=W - \xi _{w} \nabla _{W} \left(\sum \limits _{i=1}^N \ell (f(\delta '_i\odot x_i;W),t_i)\right). \end{aligned}$$

Finally, $E'$ and $W'$ are plugged into $L(E^*(A), D_e^{(\text {val})}) + \gamma L(W^*( \{\delta _i^*(E^*(A))\}_{i=1}^N ), D_a^{(\text {val})})$, resulting in $O_A=L(E', D_e^{(\text {val})}) + \gamma L(W', D_a^{(\text {val})})$. The explainer’s architecture *A* can be updated by descending the gradient of $O_A$ w.r.t *A*:

10 $$\begin{aligned} \begin{array}{l} A\leftarrow A-\eta \left( \nabla _A L(E', D_e^{(\text {val})}) +\gamma \nabla _A L(W', D_a^{(\text {val})})\right) \end{array} \end{aligned}$$

where

11 $$\begin{aligned} \begin{array}{l} \nabla _{A} L(E',A, D_e^{(\text {val})}) = \\ \nabla _{A} L(E - \xi _{e} \nabla _{E}L(E, A, D_e^{(\mathrm {tr})}),A, D_e^{(\text {val})})=\\ - \xi _{e} \nabla ^2_{A,E}L(E, A, D_e^{(\mathrm {tr})})\nabla _{E'} L(E',A, D_e^{(\text {val})})+ \nabla _{A} L(E', A, D_e^{(\text {val})}) \end{array} \end{aligned}$$

The first term in the third line involves an expensive matrix-vector product, whose computational complexity can be reduced by a finite difference approximation:

12 $$\begin{aligned} \begin{array}{ll} \nabla _{A, E}^{2} L(E,A, D_e^{(\mathrm {tr})})\nabla _{E'} L(E',A,D_e^{(\text {val})})\approx \frac{1}{2\alpha }(\nabla _{A} L( E^{+},A, D_e^{(\mathrm {tr})})-\nabla _{A} L(E^{-},A, D_e^{(\mathrm {tr})})), \end{array} \end{aligned}$$

where $E^{\pm }=E \pm \alpha \nabla _{E^{\prime }} L(E',A, D_e^{(\text {val})})$ and $\alpha$ is a small scalar that equals $0.01 /\Vert \nabla _{E'} L(E',A,D_e^{(\text {val})})\Vert _{2}$. Let $\Delta '$ denote $\{\delta '_i\}_{i=1}^N$. For $\nabla _A L(W', D_a^{(\text {val})})$ in Eq.(10), it can be calculated as

$$\begin{aligned} \frac{\partial E'}{\partial A} \frac{\partial \Delta '}{\partial E'} \frac{\partial W'}{\partial \Delta '} \nabla _{W'}L(W',D_a^{(\text {val})}), \end{aligned}$$

according to the chain rule, where

13 $$\begin{aligned} \frac{\partial W'}{\partial \Delta
'}&=\frac{\partial \Big(W - \xi _{w} \nabla _{W}\Big(\sum\nolimits_{i=1}^N \ell
(f(\delta '_i\odot x_i;W),t_i)\Big)\Big)}{\partial \Delta '} = - \xi _{w}
\nabla ^2_{\Delta ',W}\Big(\sum _{i=1}^N \ell (f(\delta '_i\odot
x_i;W),t_i)\Big), \end{aligned}$$

14 $$\begin{aligned} \frac{\partial \Delta '}{\partial
E'}&=\frac{\partial \left(\Delta - \xi _{\delta } \nabla _{\Delta
}\left(\sum\nolimits_{i=1}^N \ell (f(x_i+\delta _i;E'),f(x_i;E'))\right)\right)}{\partial
E'} = - \xi _{\delta } \nabla ^2_{E',\Delta }\left(\sum \limits _{i=1}^N
\ell (f(x_i+\delta _i;E'),f(x_i;E'))\right), \end{aligned}$$

15 $$\begin{aligned} \frac{\partial E' }{\partial A}&=\frac{\partial \Big(E - \xi _{e} \nabla _{E}L(E, A, D_e^{(\mathrm {tr})})\Big)}{\partial A} = - \xi _{e} \nabla ^2_{A,E}L\Big(E, A, D_e^{(\mathrm {tr})}\Big). \end{aligned}$$

This algorithm is summarized in Algorithm 1.
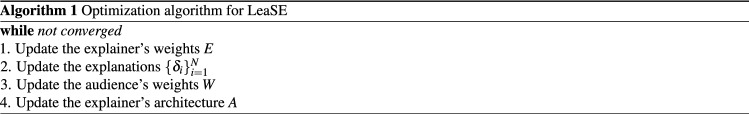


## Dataset

The data used for this work is from a public dataset^41^ on Kaggle. There are 3264 MRI images in total, which are from four classes: Glioma, Meningioma, Pituitary, and Healthy. Glioma is the most frequent type of malignant brain tumor^42^, which typically occurs in the glial cells of the brain and spinal cord. Meningioma is a type of benign brain tumor; however, it can develop into malignant tumors without proper intervention. Meningioma is typically located in meninges, which are protective membranes enclosing the brain. Like meningioma, pituitary tumors are benign and formed in the pituitary gland below the brain. Both meningioma and pituitary tumors are difficult to diagnose as they show very few symptoms. The correctness of class labels is verified by medical practitioners. The size of input images is $64\times 64$. The dataset is split into a training set with 2870 images and a test set with 394 images. Table 2 shows data split statistics. Image augmentation is performed using AutoAugment^43^.

**Table 2:** The number of training and test examples for each brain tumor type.

Brain tumor type	Number of training examples	Number of test examples
Glioma	826	100
Meningioma	822	115
Healthy	395	105
Pituitary	827	74

## Experiments

In this section, we present experimental results.

### Experimental settings

Our framework is orthogonal to existing NAS approaches and can be applied to any differentiable NAS method. In the experiments, LeaSE was applied to DARTS^12^, P-DARTS^39^, and PC-DARTS^40^. The search spaces of these methods are composed of (dilated) separable convolutions with sizes of $3\times 3$ and $5\times 5$, max pooling with size of $3\times 3$, average pooling with size of $3\times 3$, identity, and zero. Following Liu et al.^12^, each experiment consists of two phrases: 1) architecture search where an optimal cell is identified, and 2) architecture evaluation where multiple copies of the optimal cell are stacked into a larger network, which is retrained from scratch. During architecture search, the architecture of the explainer is a stack of 8 cells. Each cell consists of 7 nodes. We set the initial channel number to 16. For the audience model, we set it to ResNet-18^44^. We set the tradeoff parameter $\gamma$ to 1. We randomly split the training set into two parts. During architecture search in LeaSE, the first part is used as $D_{e}^{(\text {tr})}$ and $D_{a}^{(\text {tr})}$, and the second part is used as $D_{e}^{(\text {val})}$ and $D_{a}^{(\text {val})}$. During architecture evaluation, the composed large network is trained on the entire training set. The search algorithm was based on SGD, with a batch size of 64, an initial learning rate of 0.025 (reduced in later epochs using a cosine decay scheduler), an epoch number of 50, a weight decay of 3e-4, and a momentum of 0.9. The rest of hyperparameters mostly follow those in DARTS, P-DARTS, and PC-DARTS. During architecture evaluation, a larger network of the explainer is formed by stacking 12 copies of the searched cell. The initial channel number was set to 36. We trained the network with a batch size of 96, an epoch number of 3000, on a single Tesla v100 GPU. We compared with manually designed neural architectures including ResNet^44^, VGGNet^45^, and DenseNet^46^. We use accuracy, precision, recall, F1, specificity, area under ROC curve (AUC) as evaluation metrics.

### Results and discussion

Table 3 shows accuracy, precision, recall, F1, AUC, specificity, and the number of model parameters of different methods on the test set. From this table, we make the following observations. First, our LeaSE+DARTS method achieves the highest test accuracy, precision, recall, F1, and AUC among all methods. Its performance is much higher than ResNet and VGGNet, while its parameter size is much smaller than ResNet and VGGNet. For instance, our method achieves a test accuracy of 90.6% with 3.75M parameters while the accuracy of a human-designed network—ResNet101—is 84.5% with 42.56M parameters. As another example, our method achieves an F1 score of 91.48% with 3.75M parameters while the F1 score of another human-designed network—VGGNet16—is 89.60% with 16.03M parameters. Second, applying LeaSE to different NAS baselines including DARTS, PCDARTS, and PDARTS improves the performance of these baselines. For example, by applying LeaSE, the test accuracy of DARTS is improved from 89.34% to 90.61%, and the F1 score of PCDARTS is improved from 88.9% to 91.5%. These results strongly demonstrate the broad effectiveness of our framework in searching for better neural architectures. The reason behind this is: in our framework, explanations made by the explainer are used to train the audience model; the validation performance of the audience reflects how good the explanations are; to make good explanations, the explainer’s model must be trained well; driven by the goal of helping the audience learn well, the explainer continuously improves the training of itself. Such an explanation-driven learning mechanism is lacking in baseline methods, which are hence inferior to our method.

**Table 3:** Test accuracy (%), precision (%), recall (%), F1 (%), AUC (%), specificity (%), and the number of model parameters (millions) of different methods. DenseNet-40 denotes a DenseNet with 40 layers. Similar meanings hold for other notations in such a format.

Method	Accuracy	Precision	Recall	F1	AUC	Specificity	# Parameters (M)
DenseNet-40	83.50	86.58	81.89	84.13	91.83	92.48	0.25
DenseNet-101	86.80	89.66	86.14	87.84	92.84	96.07	0.95
VGGNet-13	88.07	90.96	88.01	89.45	94.93	98.35	10.72
VGGNet-16	88.33	91.17	88.15	89.60	94.31	98.61	16.03
ResNet-50	85.79	88.80	85.17	86.96	94.34	95.77	23.54
ResNet-101	84.52	88.72	84.47	86.53	90.06	95.20	42.56
DARTS	89.34	90.97	89.63	90.28	94.54	97.89	3.85
LeaSE+DARTS (ours)	90.61	91.49	91.50	91.48	95.60	97.99	3.75
PCDARTS	88.07	90.67	87.24	88.86	94.59	99.36	3.57
LeaSE+PCDARTS ($\gamma =0.1$, ours)	**89.60**	91.16	90.58	90.87	95.57	99.41	4.27
LeaSE+PCDARTS ($\gamma =0.5$, ours)	89.11	91.43	91.47	91.45	95.58	99.56	4.03
LeaSE+PCDARTS ($\gamma =1$, ours)	88.83	90.90	88.88	89.86	94.66	99.39	4.25
PDARTS	88.33	90.03	88.48	89.25	95.11	98.00	3.85
LeaSE+PDARTS (ours)	88.87	90.62	88.63	89.61	95.81	98.68	3.81

### Ablation studies

To better understand LeaSE, we perform an ablation study where the explainer updates its architecture by minimizing the validation loss of the audience only, without considering the validation loss of itself. Table 4 shows the results of LeaSE+DARTS and LeaSE+PDARTS. As can be seen, “audience + explainer” where the validation losses of both the audience model and the explainer itself are minimized to update the explainer’s architecture works better than “audience only” where only the audience’s validation loss is used to learn the architecture. Audience’s validation loss reflects how good the explanations made by the explainer are. Explainer’s validation loss reflects how strong the explainer’s prediction ability is. Combining these two losses provides more useful feedback to the explainer than using one loss only, which hence can help the explainer learn better.

**Table 4:** Results of the ablation study where the explainer updates its architecture by minimizing the validation loss of the audience only. “Audience Only” denotes that only the audience’s validation loss is minimized to update the architecture of the explainer. “Audience+Explainer” denotes that both the validation loss of the audience and the validation loss of the explainer are minimized to learn the explainer’s architecture. This ablation study is performed on LeaSE+DARTS and LeaSE+PDARTS. $\gamma$ is set to 1.

Method	Accuracy (%)
Audience Only on LeaSE+DARTS	90.18
Audience+Explainer on LeaSE+DARTS	90.61
Audience Only on LeaSE+PDARTS	88.49
Audience+Explainer on LeaSE+PDARTS	88.83

We also performed an ablation study on how the choice of audience models affects test accuracy. We experimented with two architectures for the audience model: ResNet-18 and VGGNet-13, where ResNet-18 is more expressive than VGGNet-13 since it has more layers. Table 5 shows the results. As can be seen, in LeaSE applied to DARTS and PDARTS, using ResNet-18 as the audience achieves better performance than using VGGNet-13. The reason is: to help a stronger audience learn better, the explainer must be even stronger. For a stronger audience model, it already has great capability in achieving excellent classification performance. To further improve this audience, explanations used to train this audience need to be very sensible and informative. To generate such explanations, the explainer has to force itself to learn very well.

**Table 5:** Results on how different choices of audience models affect test accuracy. LeaSE+DARTS+VGGNet13 denotes that LeaSE is applied to DARTS with VGGNet13 as an audience model. Similar meanings hold for the rest of notations in such a format.

Method	Accuracy (%)
LeaSE+DARTS+VGGNet13	90.17
LeaSE+DARTS+ResNet18	90.61
LeaSE+PDARTS+VGGNet13	88.56
LeaSE+PDARTS+ResNet18	88.83

We investigated how test accuracy changes with the tradeoff parameter $\gamma$. The third panel in Table 3 shows the results of LeaSE+PCDARTS. As can be seen, the test accuracy increases when we increase $\gamma$ from 0 (which is equivalent to vanilla PCDARTS) to 0.1. The reason is that a larger $\gamma$ enables the audience to provide stronger feedback to the explainer regarding how good the explanations are. Such feedback can guide the explainer to refine its architecture for generating better explanations. However, if $\gamma$ is further increased, the accuracy becomes worse. Under such circumstances, too much emphasis is put on evaluating how good the explanations are and less attention is paid to the predictive ability of the explainer. The architecture is biased to generating good explanations with predictive performance compromised, which leads to inferior performance.

### Visualization

**Figure 2:**
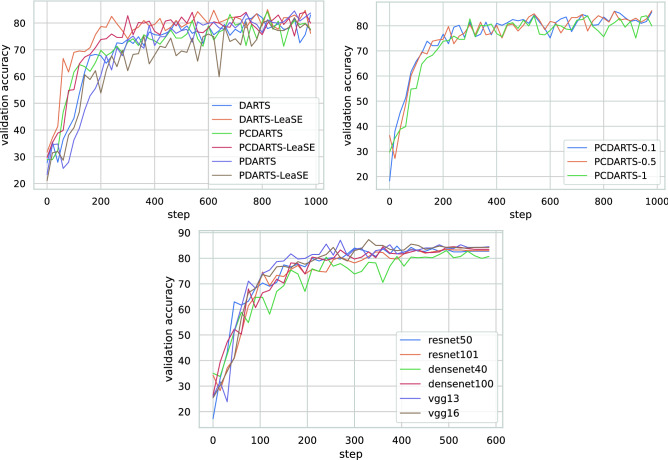
(Top left) Convergence curves of validation accuracy for different NAS methods with and without LeaSE. (Top right) Convergence curves of validation accuracy for LeaSE+PCDARTS under different $\gamma$ values. (Bottom) Convergence curves of validation accuracy for non-NAS methods.

**Figure 3:**
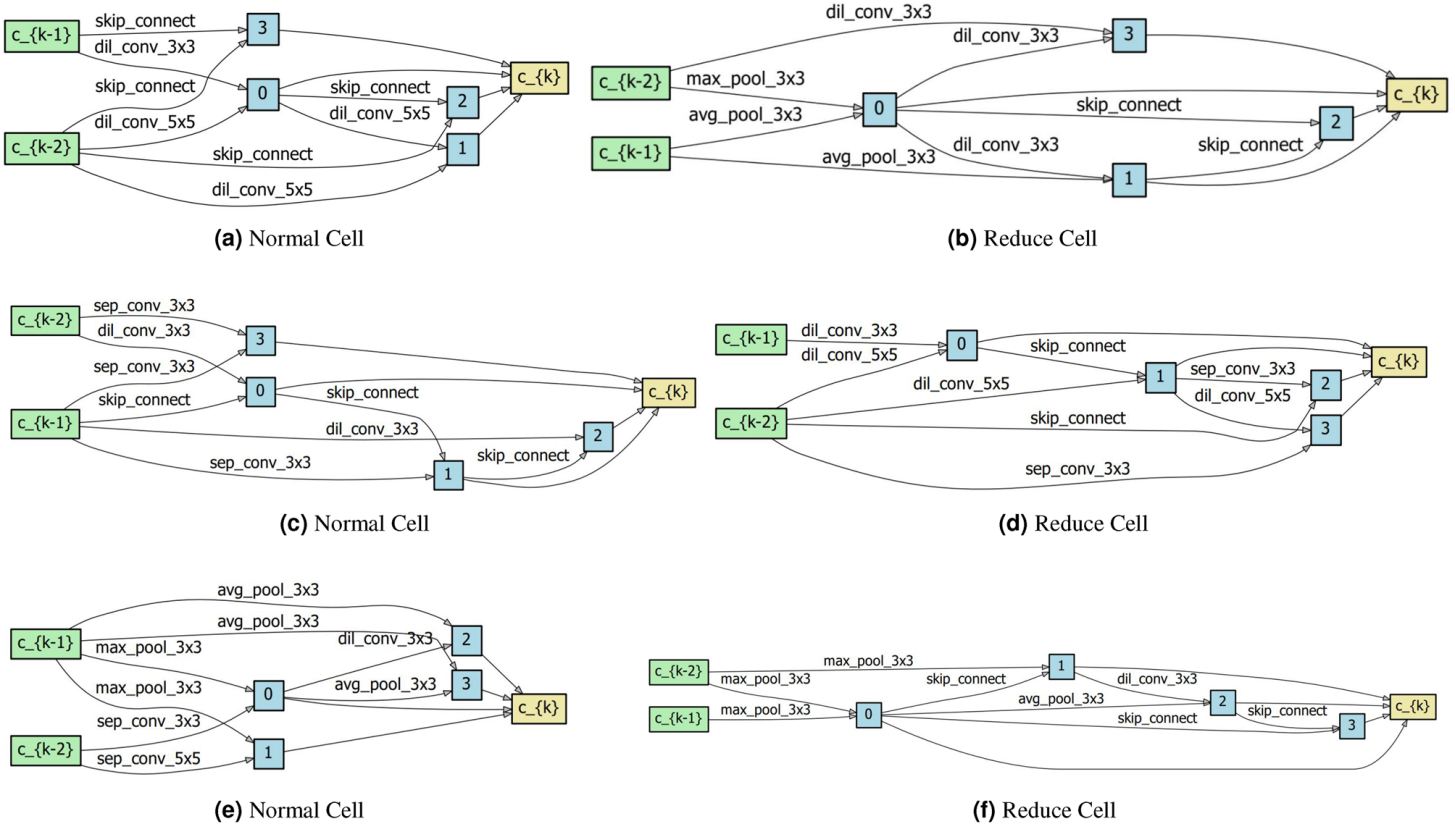
(**a, b**) Architecture searched by LeaSE+DARTS. (**c, d**) Architecture searched by LeaSE+PCDARTS. (**e, f**) Architecture searched by LeaSE+PDARTS.

We perform visualization of the results. Figure 2 shows the convergence curves of validation accuracy for different NAS methods with and without LeaSE, convergence curves of validation accuracy for LeaSE+PCDARTS under different $\gamma$ values, and convergence curves of validation accuracy for non-NAS methods. Figure 3 show the architectures searched by LeaSE+DARTS, LeaSE+PCDARTS, and LeaSE+PDARTS.

## Conclusions and future works

In this paper, we propose to automatically identify computationally efficient neural architectures that can make accurate classification of brain tumors, by leveraging a neural architecture search method—Learning by Self-Explanation (LeaSE). In LeaSE, the primary goal is to help an explainer model search for a well-performing neural architecture. The way to achieve this goal is to let the explainer make sensible explanations. The intuition behind LeaSE is that a model has to learn to understand a topic very well before it can explain this topic clearly. A four-level optimization framework is developed to formalize LeaSE, where learning is organized into four stages: the explainer learns a topic; the explainer explains this topic; the audience learns this topic based on the explanations given by the explainer; the explainer re-learns this topic based on the learning outcome of the audience. We conducted experiments on an MRI dataset with 3264 images from four classes: glioma, meningioma, pituitary tumor, and healthy. Compared with manually designed architectures, architectures searched by our methods achieve higher classification accuracy with fewer parameters. In addition, our method outperforms previous neural architecture search methods.

For future works, we plan to investigate the following directions. First, we plan to incorporate medical knowledge into our framework, such as clinical guidelines of MRI-based diagnosis and grade assessment for brain tumors, to perform knowledge-driven neural architecture search for brain tumor detection. Second, we plan to extend our framework for multi-modal brain tumor classification, by considering not only MRI images, but also other modalities, such as lab tests, medical history, vital signs, etc.

## Data Availability

The datasets generated and/or analyzed during the current study are available in the Brain Tumor Classification Dataset repository, https://www.kaggle.com/sartajbhuvaji/brain-tumor-classification-mri https://www.kaggle.com/sartajbhuvaji/brain-tumor-classification-mri .
